# Total Synthesis and Biological Activity of Marine Alkaloid Eudistomins Y_1_–Y_7_ and Their Analogues

**DOI:** 10.3390/md11051427

**Published:** 2013-04-29

**Authors:** Huijuan Jin, Puyong Zhang, Krikor Bijian, Sumei Ren, Shengbiao Wan, Moulay A. Alaoui-Jamali, Tao Jiang

**Affiliations:** 1 Key Laboratory of Marine Drugs, Chinese Ministry of Education, School of Medicine and Pharmacy, Ocean University of China, Qingdao 266003, China; E-Mails: jinhuijuan@126.com (H.J.); zhangpy819@163.com (P.Z.); rensumei@ouc.edu.cn (S.R.); biaowan@ouc.edu.cn (S.W.); 2 Departments of Medicine, Oncology, and Pharmacology and Therapeutics, Lady Davis Institute for Medical Research, Segal Cancer Center of the Sir Mortimer B. Davis Jewish General Hospital, McGill University, Montreal, QC H3T 1E2, Canada; E-Mail: krikor.bijian@mail.mcgill.ca

**Keywords:** marine alkaloid, Eudistomins Y_1_–Y_7_, β-carboline, Pictet-Spengler reaction

## Abstract

Eudistomin Y class compounds are a series of β-carbolines which was originally isolated from a marine turnicate or ascidian near the South Korea Sea. These compounds contain bromo-substituted groups, which is one of the typical characters of marine natural products. We report herein the chemical synthesis and biological evaluation of seven new β-carboline-based metabolites, Eudistomins Y_1_–Y_7_, and their hydroxyl-methylated phenyl derivatives. Using bromo-substituted tryptamines and bromo-substituted phenylglyoxals as the key intermediates, Eudistomins Y_1_–Y_7_ and their derivatives were synthesized via the acid-catalyzed Pictet-Spengler reaction and fully characterized by ^1^H- and ^13^C-NMR and mass spectroscopy. Biological studies revealed that all of the compounds showed moderate growth inhibitory activity against breast carcinoma cell line MDA-231 with IC_50_ of 15–63 μM and the inhibitory activities of hydroxyl-methylated phenyl products were higher than that of the corresponding natural products Eudistomins Y_1_–Y_7_.

## 1. Introduction

The diversity of marine compounds offers a great advantage for being developed into new drugs because of their unique and complex structures, developed through old and underexplored species evolution. However, most marine alkaloids were usually isolated in very small quantities, hindering further studies to establish their biological activities as well as structure modifications [1]. Therefore, chemical synthesis of marine alkaloids in larger quantities and by sufficient means is necessary to investigate their mode of action and their biological implications. β-carbolines are a large group of natural and synthetic indole alkaloids with different substitutions at the C-1, C-6 and C-7 position, some of which are widely distributed in many plants and mammals and exhibit a wide spectrum of biological activities [2,3,4].

Eudistomin Y class β-carboline compounds are different from previously isolated marine metabolites due to the presence of a benzoyl group attached to the β-carboline nucleus at C-1. These compounds contain bromo-substituted groups, which is one of the typical characters of the marine natural products. Seven eudistomins compounds, Eudistomins Y_1_–Y_7_, were isolated from a tunicate of the genus *Eudistoma* collected near Tong-Yeong City, South Sea, Korea in 2008 [5], and another six Eudistomins Y_8_–Y_13_ were isolated from Korean ascidian *Synoicum* sp. in 2012 [6]. From bioactivity evaluation results, several of these natural compounds exhibited moderate to significant antibacterial, antimicrobial activity and weak cytotoxic activity [5,6].

Kennedy *et al*. have reported the synthetic methods of Eudistomins Y_1_–Y_7_ with 3 steps in a two-pot process; overall yields ranged from 6% to 25% [7]. In the present paper, we report the total chemical syntheses of the marine alkaloid Eudistomins Y_1_–Y_7_ **23**–**29** and their hydroxyl-methylated phenyl derivatives (**16**–**22**) with modified one-pot oxidation via the acid-catalyzed Pictet-Spengler reaction, as well as their biological activities against breast carcinoma cell line MDA-231. All structures of target compounds were confirmed by ^1^H NMR, ^13^C NMR and HRMS. The spectra data of synthetic compounds **23**–**29** were consistent with those of natural β-carboline alkaloids Eudistomins Y_1_–Y_7_ in the literature [5].

## 2. Results and Discussion

### 2.1. Chemistry

**Scheme 1:**
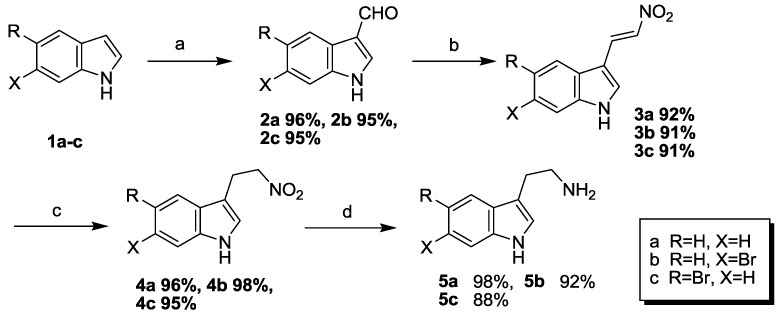
Reagents and conditions: (**a**) POCl_3_/DMF/NaOH; (**b**) CH_3_NO_2_/CH_3_COONH_4_/benzene, reflux; (**c**) NaBH_4_/THF/CH_3_OH, rt.; (**d**) Pd/C, H_2_, rt., for **5a**; LiAlH_4_/THF, refulx, for **5b** and **5c**.

The particularity of the marine environment leads to the rich bromine in the marine natural products, so the syntheses of bromo-substituted indole compounds are the key step in the preparations of marine alkaloid Eudistomins Y_1_–Y_7_ **23**–**29**. The target compounds were started from the commercially available indole. Substituted indole-3-carboxaldehyde **2a**–**c** was synthesized from **1a**–**c** by Vilsmeier-Hacck reaction with phosphorus oxychloride (POCl_3_) and *N*,*N*-dimethylformamide (DMF) according to the reference [8]. Intermediates **2a**–**c** was condensed with nitromethane (CH_3_NO_2_) based on the classical Henry reaction to form the corresponding vinyl nitro compounds **3a**–**c** [9], reduction of which by NaBH_4_ at room temperature gave compounds **4a**–**c** in high yields. Subsequently, **4a** was reduced by Pd/C in methanol at room temperature to give **5a**. **4b**–**c** were reduced by LiAlH_4_ and refluxed in THF to give bromo-substituted tryptamines **5b**–**c** [10]. The reaction routes were outlined in Scheme 1.

**Scheme 2:**
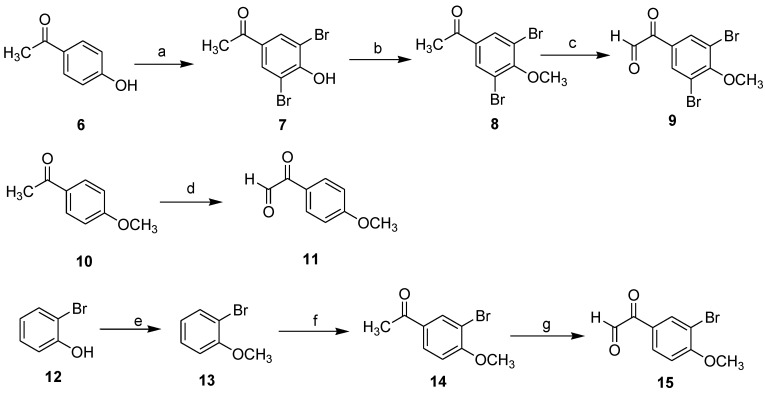
Reagents and conditions: (**a**) NBS/H_2_SO_4_/H_2_O, 92%; (**b**) (CH_3_)_2_SO_4_/K_2_CO_3_/acetone, 95%; (**c**) SeO_2_/dioxane, 80 °C, 71%; (**d**) SeO_2_/dioxane, 80 °C, 75%; (**e**) (CH_3_)_2_SO_4_/K_2_CO_3_/acetone; (**f**) acetyl chloride, AlCl_3_/CS_2_, 76%; (**g**) SeO_2_/dioxane, 80 °C, 74%.

Methylation of bromophenol by dimethyl sulfate in acetone, **7** and **12** was converted to substituted methoxybenzene **8** and **13**. Intermediate **14** was prepared by 1-bromo-2-methoxybenzene **13** using acetyl chloride by Friedel–Craft acylation. The substituted acetophenones **8**, **10** and **14** were converted to substituted phenylglyoxals **9**, **11** and **15** by oxidation with SeO_2_ in dioxane–water mixture [11]. The reaction routes were outlined in Scheme 2.

**Scheme 3:**
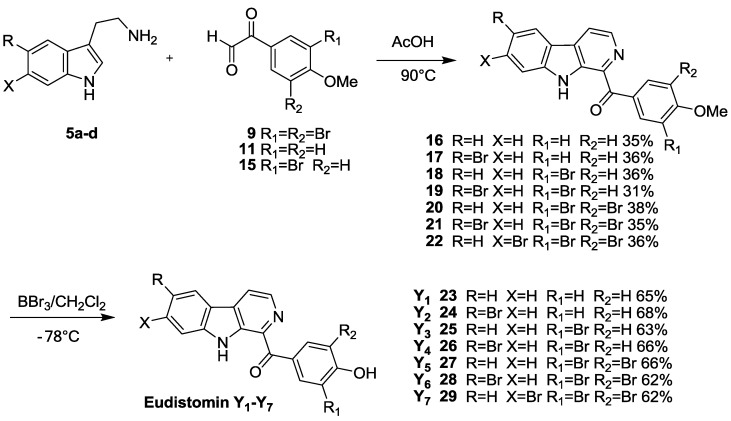
The synthesis routes of Eudistomins Y_1_–Y_7_.

The classical method to prepare β-carboline alkaloids through Pictet-Spengler reaction was a two-step method, and involved the acid-catalyzed condensation of an aliphatic amine attached to a sufficiently reactive aromatic nucleus with aldehydes. Specifically, in the first step, an imine was formed, which can be activated by acids, while in the second step, the *endo* cyclization occurred between a carbon nucleophile of a sufficiently reactive aromatic moiety and the activated iminium ion, resulting in tetrahydro-β-carboline by the formation of *N*-heterocyclic ring through a new C–C bond. After dehydrogenation, tetrahydro-β-carboline was converted to β-carboline [12,13,14,15]. In our experiments, the treatment of substituted tryptamines with substituted phenylglyoxal [16,17] under the acidic conditions did not produce the expected tetrahydro-β-carboline but rather directly generated a dehydrogenated β-carboline product **16**–**22** as reported by Zhang *et al*. [18]. It is possible that the acidity and polarity of glacial acetic acid promote the reaction of cyclization and dehydrogenation aromatizing to completion continuously. The reaction routes were outlined in Scheme 3. Pictet-Spengler cyclization of substituted tryptamine **5a**–**d** with substituted phenylglyoxal **9**, **11** or **15** in acetic acid afforded compounds **16**–**22**, which was transformed into the target compounds Eudistomins Y_1_–Y_7_ **23**–**29** in the presence of BBr_3_ in CH_2_Cl_2_ at −1278 °C with the yields of 62%–68%. The reaction routes were outlined in Scheme 3.

The modified one-pot oxidation reaction shown as the Scheme 3 was more efficient and convenient in preparing 1-substituted β-carbolines without the need of aromatization step or decarboxylation. Using this modified Pictet-Spengler reaction, the target compounds Eudistomins Y_1_–Y_7_ **23**–**29** were synthesized.

### 2.2. Biological Results and Discussion

The *in vitro* anti-proliferative activity of select compounds **16**–**29 **was evaluated against the breast carcinoma cell line MDA-231 using the 3-(4,5-dimethylthiazo-2-yl)-2,5-diphenyltetrazolium bromide (MTT) metabolic assay. Briefly, exponentially growing cells (1 × 10^3^ cells) were seeded in 96-well plates. After 18 h, cells were continuously treated with compounds **16**–**29**. Following this, after 96 h, cell survival was evaluated. The inhibitory activity (IC_50_) of the various compounds on cell proliferation was determined (Table 1). Moderate anti-proliferative activity was observed with all the compounds tested. Surprisingly, Eudistomins Y_1_–Y_3_ and Y_5_–Y_7_ with a hydroxyl group were found to have a poor cytotoxic activity compared with their precursors **16**–**18** and **20**–**22**. In summary, *in vitro* inhibitory activities of methylated products were preferable to the demethylated products except for compounds **19** and **26**.

**Table 1:** Cytotoxicity of eudistomins Y_1_–Y_7_ **23**–**29** and derivatives **16**–**22** (Scheme 3) *in vitro* ^a^ (μM).

Compound	IC_50_ (μM) ^b^	Compound	IC_50_ (μM) ^b^
	MDA-231 ^c^		MDA-231 ^c^
**16**	31.1	**23**	56.3
**17**	15.8	**24**	51.1
**18**	30.2	**25**	63.6
**19**	31.2	**26**	15.6
**20**	28.1	**27**	32.1
**21**	20.1	**28**	28.1
**22**	25.9	**29**	37.2

^a^ Data represent the mean values of three independent determinations; ^b^ Cytotoxicity as IC_50_ for each cell line, is the concentration of compound that causes a 50% growth inhibition to untreated cells using the MTT assay; ^c^ Breast carcinoma cell lines MDA-231.

In order to determine the intracellular target of these compounds, the inhibitory activity of compound **26** was tested against various kinases *in vitro*. Briefly, human recombinant full-length kinases were incubated in kinase buffer containing ATP and substrate (Poly Glu:Tyr) for 4 h at room temperature with or without the presence of compounds **26** at 10 μM final concentration. Remaining ATP in solution was then quantified utilizing the Kinase-Glo-luminescence kit (Promega). The ability of compound **26** to inhibit the kinase activity of these kinases was evaluated (Table 2). As such, the anti-proliferative activity of compound **26** does not seem to be significantly modulated by any of the kinases tested.

**Table 2:** Inhibitory kinase activity of compound **26** (10 μM) against various kinases.

Kinase	Aurora B	EGFR	MEK1	FAK	Src
Inhibition (%)	10	0	3	9	2

## 3. Experimental Section

### 3.1. Materials and Methods

The starting materials and reagents, purchased from commercial suppliers, were used without further purification. All reactions were monitored by thin-layer chromatography (TLC), on aluminium sheets (Silica gel 60-F_254_, E. Merck). Compounds were visualized by UV light. Column chromatography was carried out using silica gel (200–300 mesh). All reaction solvents were dried prior to use according to standard procedures. All primary reagents were commercially available. Silica gel chromatography solvents were of analytical grade. Melting points were recorded on a micro melting point apparatus MP-500D and were uncorrected. NMR spectra were recorded on a Jeol JNM-ECP spectrometer at 600 MHz for ^1^H NMR and 150 MHz for ^13^C NMR with TMS as the internal standard. Chemical shifts are expressed in δ (ppm) and coupling constants (*J*) in Hz. Multiplicity is indicated as follows: s (singlet), d (doublet), t (triplet), dd (doublet of doublets), brs (broad singlet), *etc*. Mass spectra were recorded using a Q-TOF Ultima™ Global by chemical ionization.

### 3.2. General Procedure for Compounds **2a–c**

In a separate tear-shaped flask under a nitrogen atmosphere, 40 mL of DMF was cooled to 0 °C, and then POCl_3_ (150 mmol) was added dropwise within 0.5 h under nitrogen. After the addition, stirring was continued for 1.5 h at 0 °C. It was followed by addition of a solution of indole **1a**–**c** (100 mmol) in DMF (20 mL) below 0 °C within 1.5 h. Then the reaction solution was heated to 40 °C for another 2 h and cooled to 0 °C. Subsequently, crushed ice (50 g) was added, and then the pH was adjusted to 8–9 by adding NaOH solution (20%, w/w). The resulting reaction mixture was then heated at reflux for 12 h. And then, the resulting suspension solution was cooled naturally to the room temperature and filtered through celite to afford a white crude product. The crude product was purified by flash column chromatography using silica gel as the stationary phase and using ethyl acetate/hexane (1:3) as the mobile phase to provide the compounds **2a**–**c** [8].

1*H*-Indole-3-carbaldehyde (**2a**): White solid; yield 96%; ^1^H NMR (600 MHz, CDCl_3_) δ 11.21 (brs, 1H), 10.04 (s, 1H), 8.24 (d, *J* = 7.8 Hz, 1H), 8.21 (s, 1H), 7.54 (d, *J* = 7.8 Hz, 1H), 7.30–7.23 (m, 2H); ^13^C NMR (151 MHz, CDCl_3_) δ 184.6, 137.3, 124.7, 123.7, 122.2, 121.4, 119.2, 112.2.

6-Bromo-1*H*-indole-3-carbaldehyde (**2b**): White solid; yield 95%; ^1^H NMR (600 MHz, CDCl_3_) δ 11.33 (brs, 1H), 10.03 (s, 1H), 8.24 (s, 1H), 8.15 (d, *J* = 8.3 Hz, 1H), 7.76 (d, *J* = 1.8 Hz, 1H), 7.39 (dd, *J* = 8.3, 1.8 Hz, 1H); ^13^C NMR (151 MHz, CDCl_3_) δ 184.7, 137.9, 125.3, 123.6, 122.9, 119.0, 116.5, 115.2.

5-Bromo-1*H*-indole-3-carbaldehyde (**2c**): White solid; yield 95%; ^1^H NMR (600 MHz, CDCl_3_) δ 11.36 (brs, 1H), 10.02 (s, 1H), 8.39 (d, *J* = 1.8 Hz, 1H), 8.26 (s, 1H), 7.53 (d, *J* = 8.7 Hz, 1H), 7.40 (dd, *J* = 8.7 Hz, 1.8 Hz, 1H); ^13^C NMR (151 MHz, CDCl_3_) δ 184.6, 138.2, 136.1, 126.5, 126.4, 123.8, 118.5, 115.3, 114.2.

### 3.3. General Procedure for Compounds **3a–c**

To a solution of 1*H*-indole-3-carbaldehyde **2a**–**c** (80 mmol) in CH_3_NO_2 _(80 mL), was added ammonium acetate (40 mmol) and benzene (1.1 mL, 12.4 mmol). The reaction mixture was heated at reflux for 12 h. After cooling to room temperature, some solvent was removed under reduced pressure and filtered through Celite to afford a yellow crude product. The crude product was purified by flash column chromatography using silica gel as the stationary phase and using ethyl acetate/hexane (1:2) as the mobile phase to provide the compounds **3a**–**c** [9].

(*E*)-3-(2-Nitrovinyl)-1*H*-indole (**3a**): Deep yellow solid; yield 92%; ^1^H NMR (600 MHz, CDCl_3_) δ 11.33 (brs, 1H), 8.39 (d, *J* = 13.3 Hz, 1H), 8.16 (d, *J* = 3.2 Hz, 1H), 7.97 (dd, *J* = 5.9, 2.3 Hz, 1H), 7.92 (d, *J* = 13.3 Hz, 1H), 7.59 (dd, *J* = 5.9, 2.3 Hz, 1H), 7.32–7.29 (m, 2H); ^13^C NMR (151 MHz, CDCl_3_) δ 138.2, 135.1, 133.9, 131.9, 125.1, 123.6, 122.1, 120.5, 112.8, 108.8.

(*E*)-6-Bromo-3-(2-nitrovinyl)-1*H*-indole (**3b**): Deep yellow solid; yield 91%; ^1^H NMR (600 MHz, CDCl_3_) δ 11.39 (brs, 1H), 8.36 (d, *J* = 13.3 Hz, 1H), 8.18 (s, 1H), 7.95 (d, *J* = 8.3 Hz, 1H), 7.91 (d, *J* = 13.3 Hz, 1H), 7.78 (d, *J* = 1.8 Hz, 1H), 7.41 (dd, *J* = 8.3, 1.8 Hz, 1H); ^13^C NMR (151 MHz, CDCl_3_) δ 138.9, 135.4, 133.2, 132.7, 124.9, 124.1, 122.0, 116.5, 115.7, 108.8.

(*E*)-5-Bromo-3-(2-nitrovinyl)-1*H*-indole (**3c**): Deep yellow solid; yield 91%; ^1^H NMR (600 MHz, CDCl_3_) δ 11.41 (brs, 1H), 8.35 (d, *J* = 13.7 Hz, 1H), 8.19 (s, 1H), 8.16 (d, *J* = 1.8 Hz, 1H), 7.97 (d, *J* = 13.7 Hz, 1H), 7.54 (d, *J* = 8.7 Hz, 1H), 7.42 (dd, *J* = 8.7, 1.8 Hz, 1H); ^13^C NMR (151 MHz, CDCl_3_) δ 136.7, 135.5, 133.0, 132.7, 126.8, 126.3, 122.9, 115.0, 114.5, 108.3.

### 3.4. General Procedure for Compounds **4a–c**

To a solution of (*E*)-3-(2-nitrovinyl)-1*H*-indole **3a**–**c** (20mmol) in THF (60 mL) and CH_3_OH (9 mL), was added NaBH_4_ (40 mmol) in batch over 0.5 h. The above reaction solution was stirred at room temperature for about 1 h and the completion of the reaction was monitored by TLC. Then water (100 mL) and hydrochloric acid (100 mL, 10%, v/v) was added slowly. The resulting reaction mixture was extracted with CH_2_Cl_2_ (30 mL × 3). The combined organic phase was washed with H_2_O (20 mL × 3) and brine (20 mL × 3), dried over anhydrous MgSO_4_ and the solvent was removed under reduced pressure. The residue was purified by flash column chromatography using silica gel as the stationary phase and using ethyl acetate/hexane (1:3) as the mobile phase to provide the compounds **4a**–**c** [10].

3-(2-Nitroethyl)-1*H*-indole (**4a**): Brown solid; yield 96%; ^1^H NMR (600 MHz, CDCl_3_) δ 8.09 (brs, 1H), 7.59 (d, *J* = 7.8 Hz, 1H), 7.38 (d, *J* = 8.3 Hz, 1H), 7.25 (t, *J* = 7.8, 7.3 Hz, 1H), 7.18 (t, *J* = 8.3, 7.3 Hz, 1H), 7.04 (s, 1H), 4.67 (t, *J* = 6.9 Hz, 2H), 3.49 (t, *J* = 6.9 Hz, 2H); ^13^C NMR (151 MHz, CDCl_3_) δ 136.3, 126.7, 122.7, 122.6, 119.9, 118.2, 111.6, 110.0, 75.8, 23.7.

6-Bromo-3-(2-nitroethyl)-1*H*-indole (**4b**): Brown solid; yield 98%; ^1^H NMR (600 MHz, CDCl_3_) δ 8.13 (brs, 1H), 7.49 (d, *J* = 1.8 Hz, 1H), 7.41 (d, *J* = 8.2 Hz, 1H), 7.24 (dd, *J* = 8.2, 1.8 Hz, 1H), 7.00 (d, *J* = 1.8 Hz, 1H), 4.64 (t, *J* = 6.9 Hz, 2H), 3.49 (t, *J* = 6.9 Hz, 2H); ^13^C NMR (125 MHz, CDCl_3_) δ 137.1, 125.7, 123.3, 123.2, 119.5, 116.1, 114.5, 110.3, 75.8, 23.5.

5-Bromo-3-(2-nitroethyl)-1*H*-indole (**4c**): Brown solid; yield 95%; ^1^H NMR (600 MHz, CDCl_3_) δ 8.18 (brs, 1H), 7.68 (d, *J* = 1.8 Hz, 1H), 7.29 (dd, *J* = 8.7, 1.8 Hz, 1H), 7.22 (d, *J* = 8.7 Hz, 1H), 7.03 (d, *J* = 1.8 Hz, 1H), 4.64 (t, *J* = 7.3 Hz, 2H), 3.41 (t, *J* = 7.3 Hz, 2H); ^13^C NMR (151 MHz, CDCl_3_) δ 134.9, 128.5, 125.4, 124.0, 120.8, 113.2, 113.1, 109.7, 75.7, 23.4.

2-(1*H*-indol-3-yl)ethanamine (**5a**): To a solution of 3-(2-nitroethyl)-1*H*-indole **4a** (1.9 g, 10 mmol) in methanol (30 mL), was added 10% Pd/C (0.08 g). H_2_ was passed in at room temperature, then hydrogenated under normal pressure for 24 h. The completion of the reaction was monitored by TLC. The reaction mixture was then filtered and evaporated under reduced pressure. The residue was purified by flash column chromatography using silica gel as the stationary phase and using ethyl acetate/methanol (10:1) as the mobile phase to provide 2-(1*H*-indol-3-yl)ethanamine **5a** as an white solid in a yield of 98%. ^1^H NMR (600 MHz, CDCl_3_) δ 11.04 (brs, 1H), 7.57 (d, *J* = 7.8 Hz, 1H), 7.42 (d, *J* = 7.8 Hz, 1H), 7.17 (s, 1H), 7.11 (t, *J* = 7.8 Hz, 1H), 7.02 (t, *J* = 7.8 Hz, 1H), 2.89 (t, *J* = 6.8 Hz, 2H), 2.83 (t, *J* = 6.8 Hz, 2H), 2.70 (s, 2H); ^13^C NMR (151 MHz, CDCl_3_) δ 136.9, 127.9, 123.2, 121.4, 118.9, 113.0, 111.9, 43.1, 29.8.

### 3.5. General Procedure for Compounds **5b–c**

To a solution of substituted 3-(2-nitroethyl)-1*H*-indole **4b**–**c** (10 mmol) in THF (30 mL), was added LiAlH_4_ (20 mmol) slowly. After the addition, it was heated at reflux for about 5 h. The completion of the reaction was monitored by TLC. The mixture was allowed to cool to room temperature and quenched by dropwise addition of saturated Na_2_SO_4_ solution. The resulting suspension mixture was filtered through Celite, and the filtrate was extracted with EtOAc (30 mL × 3). The combined organic phase was washed with H_2_O (20 mL × 3) and brine (20 mL × 3), dried over anhydrous MgSO_4_ and the solvent was removed under reduced pressure. The residue was purified by flash column chromatography using silica gel as the stationary phase and using ethyl acetate/methanol (10:1) as the mobile phase to provide the compounds **5b**–**c** as a gray solid [10].

2-(6-Bromo-1*H*-indol-3-yl)ethanamine (**5b**): White solid; yield 92%; ^1^H NMR (600 MHz, CDCl_3_) δ 11.08 (brs, 1H), 7.52 (d, *J* = 1.8 Hz, 1H), 7.47 (d, *J* = 8.7 Hz, 1H), 7.18 (s, 1H), 7.09 (dd, *J* = 8.7, 1.8 Hz, 1H), 3.53 (s, 2H), 2.83 (t, *J* = 6.4 Hz, 2H), 2.77 (t, *J* = 6.4 Hz, 2H); ^13^C NMR (151 MHz, CDCl_3_) δ 137.7, 126.9, 124.4, 121.5, 120.7, 114.5, 114.2, 113.1, 42.6, 28.7.

2-(5-Bromo-1*H*-indol-3-yl)ethanamine (**5c**): White solid; yield 88%; ^1^H NMR (600 MHz, CDCl_3_) δ 11.17 (brs, 1H), 7.75 (d, *J* = 1.8 Hz, 1H), 7.31–7.21 (m, 2H), 7.16 (s, 1H), 3.51 (s, 2H), 2.83 (t, *J* = 6.4 Hz, 2H), 2.74 (t, *J* = 6.4 Hz, 2H); ^13^C NMR (151 MHz, CDCl_3_) δ 135.4, 129.7, 124.9, 124.0, 123.7, 121.1, 113.9, 111.2, 42.4, 28.5.

1-(3,5-Dibromo-4-hydroxyphenyl)ethanone (**7**): To a suspended mixture of 1-(4-hydroxyphenyl)ethanone (4.08 g, 30 mmol) and deionized water (200 mL), was added *N*-Bromosuccinimide (8.01 g, 45 mmol). The reaction mixture was heated to 60 °C, then 40% (v/v) H_2_SO_4_ (20 mL) was added. After stirred for about 10 h and the completion of the reaction was monitored by TLC, the reaction mixture was filtered through Celite to afford a white crude product. The crude product was purified by flash column chromatography using silica gel as the stationary phase and using ethyl acetate/hexane (1:3) as the mobile phase to provide a compound 1-(3,5-dibromo-4-hydroxyphenyl)ethanone (**7**) as a white solid in the yield of 92%.

1-(3,5-Dibromo-4-methoxyphenyl)ethanone (**8**): To a solution of **7** (20 mmol) in anhydrous acetone (50 mL) were added potassium carbonate (30.0 mmol) and dimethyl sulfate (2.84 mL, 30.0 mmol), and refluxed for 2 h. After cooled to room temperature, the reaction mixture was filtered through Celite, and the filtrate was concentrated under vacuum. The residue was purified by flash column chromatography using silica gel as the stationary phase and using ethyl acetate/hexane (1:2) as the mobile phase to provide the desired compound **8** as a white solid in the yield of 95%. ^1^H NMR (600 MHz, CDCl_3_) δ 8.08 (s, 2H), 3.93 (s, 3H), 2.56 (s, 3H); ^13^C NMR (151 MHz, CDCl_3_) δ 194.5, 158.1, 135.1, 133.0, 118.7, 60.9, 26.6.

### 3.6. General Procedure for Compounds **9**, **11**, **15**

To a solution of dioxane/deionized water (20:1) (30 mL) was added SeO_2_ (12 mmol). The mixture was heated to 80 °C and stirred until the solid dissolved. It was followed by addition of substituted phenylethanone **8**, **10**, **14** (10 mmol), and was refluxed for about 12 h and the completion of the reaction was monitored by TLC. The hot solution was filtered through Celite, and the filtrate was concentrated under vacuum. The residue was purified by flash column chromatography using silica gel as the stationary phase and using ethyl acetate/hexane (1:3) as the mobile phase to provide the desired compound **9**, **11**, **15** [11].

2-(3,5-Dibromo-4-methoxyphenyl)-2-oxoacetaldehyde (**9**): Light brown solid; yield 71%; ^1^H NMR (600 MHz, CDCl_3_) δ 9.42 (s, 1H), 8.28 (s, 1H), 8.22 (s, 1H), 3.85 (s, 3H); ^13^C NMR (151 MHz, CDCl_3_) δ 193.6, 191.9, 157.9, 134.5, 132.3, 118.3, 61.2.

2-(4-Methoxyphenyl)-2-oxoacetaldehyde (**11**): Light brown solid; yield 75%; ^1^H NMR (600 MHz, CDCl_3_) δ 9.53 (s, 1H), 8.06 (d, *J* = 8.7 Hz, 2H), 7.02 (d, *J* = 8.7 Hz, 2H), 3.83 (s, 3H); ^13^C NMR (151 MHz, CDCl_3_) δ 195.2, 192.5, 164.0, 132.4, 126.7, 114.4, 56.1.

2-(3-Bromo-4-methoxyphenyl)-2-oxoacetaldehyde (**15**): Light brown solid; yield 74%; ^1^H NMR (600 MHz, CDCl_3_) δ 9.43 (s, 1H), 8.28 (s, 1H), 8.09 (d, *J* = 8.7 Hz, 1H), 7.21 (d, *J* = 8.7 Hz, 1H), 3.93 (s, 3H); ^13^C NMR (151 MHz, CDCl_3_) δ 194.4, 192.6, 159.9, 134.8, 131.7, 127.8, 112.7, 110.9, 57.2.

1-(3-Bromo-4-methoxyphenyl)ethanone (**14**): To a suspension mixture of anhydrous AlCl_3_ (3.0 g, 22.5 mmol) in CS_2_ (50 mL) , was added 1-bromo-2-methoxybenzene (2.79 g, 15 mmol). The above mixture was maintained at room temperature and stirred for 0.5 h, and then acetyl chloride (1.6 mL, 22.5 mol) was added dropwise. After the addition, stirring was continued for another 0.5 h. And then, the reaction mixture was refluxed for 2 h. After cooled to room temperature, it was poured into 80 mL of ice-water containing 20 mL of concentrated hydrochloric acid and stirred to reach room temperature. The mixture was extracted with CH_2_Cl_2_ (20 mL × 3). The combined organic phase was washed with H_2_O (20 mL × 3), 10% aqueous NaOH (10 mL × 2), H_2_O (20 mL × 3) and brine (20 mL × 3), dried over anhydrous MgSO_4_ and the solvent was removed under reduced pressure. The residue was purified by recrystallization from petroleum ether (20 mL) to afford 1-(3-bromo-4-methoxyphenyl)ethanone (**14**) as a brown solid in the yield of 76%.

### 3.7. General Procedure for Compounds **16–22**

To a solution of substituted phenylglyoxal **9**, **11**, **15** (3 mmol) in AcOH (30 mL), was added substituted tryptamine **5a**–**d** (3 mmol). The above mixture was heated at 90 °C for 10 h, then cooled and adjusted pH to 5 by adding concentrated ammonium hydroxide. The resulting mixture was diluted with 100 mL of water and extracted with EtOAc (30 mL × 3). The combined organic phase was washed with H_2_O (20 mL × 3) and brine (20 mL × 3), dried over anhydrous MgSO_4_ and the solvent was removed under reduced pressure. The residue was purified by flash column chromatography using silica gel as the stationary phase and using ethyl acetate/hexane (1:3) as the mobile phase to provide the desired compounds **16**–**22**.

(4-Methoxyphenyl)(9*H*-pyrido[3,4-*b*]indol-1-yl)methanone (**16**): Yellow solid; yield 35%; mp 182–183 °C; ^1^H NMR (600 MHz, CDCl_3_) δ 10.46 (brs, 1H), 8.60 (d, *J* = 5.0 Hz, 1H), 8.45 (d, *J* = 8.7 Hz, 2H), 8.17 (d, *J* = 7.8 Hz, 1H), 8.15 (d, *J* = 5.0 Hz, 1H), 7.62–7.58 (m, 2H), 7.34 (t, *J* = 7.8 Hz, 1H), 7.03 (d, *J* = 8.7 Hz, 2H), 3.91 (s, 3H); ^13^C NMR (151 MHz, CDCl_3_) δ 193.7, 163.4, 141.1, 138.0, 137.4, 137.1, 134.0, 131.7, 130.4, 129.4, 122.0, 121.0, 120.8, 118.4, 113.6, 112.1, 55.7; HRMS: *m/z* calcd. for C_19_H_15_N_2_O_2_^+^, 303.1133; found: 303.1130.

(6-Bromo-9*H*-pyrido[3,4-*b*]indol-1-yl)(4-methoxyphenyl)methanone (**17**): Yellow solid; yield 36%; mp 194–195 °C; ^1^H NMR (600 MHz, CDCl_3_) δ 10.48 (brs, 1H), 8.62 (d, *J* = 5.0 Hz, 1H), 8.45 (d, *J* = 8.7 Hz, 2H), 8.29 (d, *J* = 1.8 Hz, 1H), 8.10 (d, *J* = 5.0 Hz, 1H), 7.68 (dd, *J* = 8.7, 1.8 Hz, 1H), 7.48 (d, *J* = 8.7 Hz, 1H), 7.04 (d, *J* = 8.7 Hz, 2H), 3.92 (s, 3H); ^13^C NMR (151 MHz, CDCl_3_) δ 193.4, 163.5, 139.7, 138.3, 137.6, 137.4, 134.0, 132.1, 130.5, 130.1, 124.7, 122.8, 120.8, 118.5, 113.6, 113.5, 55.7; HRMS: *m/z* calcd. for C_19_H_14_N_2_O_2_Br^+^, 381.0239; found: 381.0237.

(3-Bromo-4-methoxyphenyl)(9*H*-pyrido[3,4-*b*]indol-1-yl)methanone (**18**): Yellow solid; yield 36%; mp 192–193 °C; ^1^H NMR (600 MHz, CDCl_3_) δ 10.40 (brs, 1H), 8.69 (d, *J* = 1.8 Hz, 1H), 8.59 (d, *J* = 5.0 Hz, 1H), 8.49 (dd, *J* = 8.7, 2.2 Hz, 1H), 8.15 (m, 2H), 7.60 (m, 2H), 7.35–7.33 (m, 1H), 7.01 (d, *J* = 8.7 Hz, 1H), 3.99 (s, 3H); ^13^C NMR (151 MHz, CDCl_3_) δ 192.2, 159.4, 141.1, 138.1, 137.5, 136.9, 136.5, 133.1, 131.8, 131.4, 129.5, 122.0, 120.9, 118.7, 112.2, 111.5, 110.9, 56.6; HRMS: *m/z* calcd. for C_19_H_14_N_2_O_2_Br^+^, 381.0239; found: 381.0220.

(3-Bromo-4-methoxyphenyl)(6-bromo-9*H*-pyrido[3,4-*b*]indol-1-yl)methanone (**19**): Yellow solid; yield 31%; mp 195–196 °C; ^1^H NMR (600 MHz, CDCl_3_) δ 10.50 (brs, 1H), 8.68 (d, *J* = 1.8 Hz, 1H), 8.59 (d, *J* = 5.0 Hz, 1H), 8.47 (dd, *J* = 8.7, 2.2 Hz, 1H), 8.26 (s, 1H), 8.08 (d, *J* = 5.0 Hz, 1H), 7.66 (dd, *J* = 8.7, 2.2 Hz, 1H), 7.46 (d, *J* = 8.7 Hz, 1H), 7.00 (d, *J* = 8.7 Hz, 1H), 3.99 (s, 3H); ^13^C NMR (151 MHz, CDCl_3_) δ 191.9, 159.5, 139.6, 138.3, 137.5, 136.8, 133.2, 132.2, 131.1, 130.7, 124.7, 122.7, 118.7, 113.6, 111.5, 110.9, 56.6; HRMS: *m/z* calcd. for C_19_H_13_N_2_O_2_Br_2_^+^, 458.9344; found: 458.9327.

(3,5-Dibromo-4-methoxyphenyl)(9*H*-pyrido[3,4-*b*]indol-1-yl)methanone (**20**): Yellow solid; yield 38%; mp 197–198 °C; ^1^H NMR (600 MHz, CDCl_3_) δ 10.38 (brs, 1H), 8.62 (d, *J* = 5.0 Hz, 1H), 8.59 (s, 1H), 8.20–8.18 (m, 1H), 7.65–7.62 (m, 2H), 7.38–7.36 (m, 1H), 3.98 (s, 3H); ^13^C NMR (151 MHz, CDCl_3_) δ 191.5, 157.5, 141.2, 138.4, 137.5, 136.0, 135.6, 132.1, 129.7, 122.1, 121.2, 120.9, 119.2, 118.1, 112.2, 60.9; HRMS: *m/z* calcd. for C_19_H_13_N_2_O_2_Br_2_^+^, 458.9344; found: 458.9333.

(6-Bromo-9*H*-pyrido[3,4-*b*]indol-1-yl)(3,5-dibromo-4-methoxyphenyl)methanone (**21**): Yellow solid; yield 35%; mp 234–235 °C; ^1^H NMR (600 MHz, CDCl_3_) δ 10.41 (brs, 1H), 8.64 (d, *J* = 5.0 Hz, 1H), 8.60 (s, 2H), 8.32 (d, *J* = 1.8 Hz, 1H), 8,16 (d, *J* = 5.0 Hz, 1H), 7.72 (dd, *J* = 8.7, 1.8 Hz, 1H), 7.52 (d, *J* = 8.7 Hz, 1H), 3.99 (s, 3H); ^13^C NMR (151 MHz, CDCl_3_) δ 191.3, 157.6, 139.7, 138.7, 137.6, 136.1, 135.4, 134.1, 132.5, 131.0, 124.9, 122.7, 119.3, 118.1, 114.0, 113.8, 60.9; HRMS: *m/z* calcd. for C_19_H_12_N_2_O_2_Br_3_^+^, 536.8449; found: 536.8442.

(7-Bromo-9*H*-pyrido[3,4-*b*]indol-1-yl)(3,5-dibromo-4-methoxyphenyl)methanone (**22**): Yellow solid; yield 36%; mp 216–217 °C; ^1^H NMR (600 MHz, CDCl_3_) δ 10.38 (brs, 1H), 8.63 (d, *J* = 5.0 Hz, 1H), 8.59 (s, 2H), 8,16 (d, *J* = 5.0 Hz, 1H), 8.03 (d, *J* = 8.2 Hz, 1H), 7.78 (d, *J* = 1.8 Hz, 1H), 7.48 (dd, *J* = 8.7, 1.8 Hz, 1H), 3.98 (s, 3H); ^13^C NMR (151 MHz, CDCl_3_) δ 191.3, 157.7, 141.9, 138.9, 137.5, 136.1, 135.4, 131.5, 124.7, 123.5, 123.2, 119.9, 119.1, 118.1 115.4, 60.9; HRMS: *m/z* calcd. for C_19_H_12_N_2_O_2_Br_3_^+^, 536.8449; found: 536.8464.

### 3.8. General Procedure for Compounds **23–29**

To a solution of the compounds **16**–**22** (0.5 mmol) in CH_2_Cl_2_ (10mL) at −78 °C under argon atmosphere, was slowly added dropwise BBr_3_ (5 mmol). The reaction mixture was stirred and warmed to r.t. and stirred for 24 h. NaOH solution (5 mL, 2 mol/L) was then slowly added dropwise. After addition, a short period of stirring was continued, and then the solution was acidified with hydrochloric acid (20 mL, 2 mol/L), followed by extraction with CH_2_Cl_2_ (30 mL × 3). The combined organic phase was washed with H_2_O (20 mL × 3) and brine (20 mL × 3), dried over anhydrous MgSO_4_ and the solvent was removed under reduced pressure. The residue was purified by flash column chromatography using silica gel as the stationary phase and using ethyl acetate/hexane (1:3) as the mobile phase to provide the desired compounds eudistomins Y_1_–Y_7_ **23**–**29**.

(4-Hydroxyphenyl)(9*H*-pyrido[3,4-*b*]indol-1-yl)methanone (Eudistomins Y_1_ **23**): Yellow solid; yield 65%; mp 217–218 °C; ^1^H NMR (600 MHz, acetone-*d*_6_) δ 11.30(s, 1H), 9.26 (s, 1H), 8.56 (d, *J* = 5.0 Hz, 1H), 8.47 (d, *J* = 8.8 Hz, 2H), 8.38 (d, *J* = 5.0 Hz, 1H), 8.32 (d, *J* = 7.8 Hz, 1H), 7.88 (d, *J* = 7.8 Hz, 1H), 7.62 (t,* J* = 7.8 Hz, 1H), 7. 34 (t, *J* = 7.8 Hz, 1H), 7.00 (d, *J* = 8.8 Hz, 2H); ^13^C NMR (151 MHz, acetone-*d*_6_) δ 192.0, 163.1, 142.5, 139.0, 138.4, 137.6, 135.0, 131.0, 130.2, 129.4, 122.7, 122.1, 121.3, 119.7, 115.6, 113.5; HRMS: *m/z* calcd. for C_18_H_11_N_2_O_2_^−^, 287.0821; found: 287.0830.

(6-Bromo-9*H*-pyrido[3,4-*b*]indol-1-yl)(4-hydroxyphenyl)methanone (Eudistomins Y_2_ **24**): Yellow solid; yield 68%; mp 247–248 °C; ^1^H NMR (600 MHz, acetone-*d*_6_) δ 11.46 (s, 1H), 9.30 (s, 1H), 8.60 (d, *J* = 5.0 Hz, 1H), 8.53 (d, *J* = 1.8 Hz, 1H), 8.47 (d, *J* =8.8 Hz, 2H), 8.43 (d, *J* = 5.0 Hz, 1H), 7.88 (d, *J* = 8.8 Hz, 1H), 7.76 (dd, *J* = 8.8, 1.8 Hz, 1H), 7.08 (d, *J* = 8.8 Hz, 2H); ^13^C NMR (151 MHz, acetone-*d*_6_) δ 192.2, 162.8, 141.1, 139.0, 138.5, 137.5, 135.1, 132.3, 131.1, 130.0, 125.4, 123.5, 119.1, 115.5, 113.5, 112.0; HRMS: *m/z* calcd. for C_18_H_10_N_2_O_2_Br^−^, 364.9926; found: 364.9925.

(3-Bromo-4-hydroxyphenyl)(9*H*-pyrido[3,4-*b*]indol-1-yl)methanone (Eudistomins Y_3_ **25**): Yellow solid; yield 63%; mp 231–232 °C; ^1^H NMR (600 MHz, DMSO-*d*_6_) δ 11.99 (s, 1H), 11.28 (s, 1H), 8.57 (d, *J* = 2.2 Hz, 1H), 8.54 (d, *J* = 4.4 Hz, 1H), 8.43 (d, *J* = 4.4 Hz, 1H), 8.32 (d, *J* = 7.7 Hz, 1H), 8.24 (dd, *J* = 8.8, 2.2 Hz, 1H), 7.79 (d, *J* = 8.8 Hz, 1H), 7.60 (t, *J* = 7.7 Hz, 1H), 7.31 (t, *J* = 7.7 Hz, 1H), 7.13 (d, *J* = 7.7 Hz, 1H);^ 13^C NMR (151 MHz, DMSO-*d*_6_) δ 190.1, 158.2, 141.6, 137.0, 136.7, 136.4, 135.8, 132.5, 131.0, 129.6, 128.9, 121.8, 120.1, 120.0, 118.7, 115.6, 112.9, 108.8; HRMS: *m/z* calcd. for C_18_H_10_N_2_O_2_Br^−^, 364.9926; found: 364.9932.

(3-Bromo-4-hydroxyphenyl)(6-bromo-9*H*-pyrido[3,4-*b*]indol-1-yl)methanone (Eudistomins Y_4_ **26**): Yellow solid; yield 66%; mp 265–266 °C; ^1^H NMR (600 MHz, DMSO-*d*_6_) δ 12.13 (s, 1H), 11.30 (s, 1H), 8.61 (s, 1H), 8.57 (d, *J* = 5.5 Hz, 1H), 8.56 (s, 1H), 8.50 (d, *J* = 5.5 Hz, 1H), 8.23 (dd, *J* = 8.8, 2.2 Hz, 1H), 7.75–7.72 (m, 1H), 7.13 (d, *J* = 8.8 Hz, 1H); ^13^C NMR (151 MHz, DMSO-*d*_6_) δ 189.9, 158.3, 140.3, 137.3, 137.1, 136.4, 135.9, 132.5, 131.4, 129.9, 129.4, 124.5, 122.0, 119.1, 115.6, 114.9, 112.2, 108.8; HRMS: *m/z* calcd. for C_18_H_9_N_2_O_2_Br_2_^−^, 442.9031; found: 442.9012.

(3,5-Dibromo-4-hydroxyphenyl)(9*H*-pyrido[3,4-*b*]indol-1-yl)methanone (Eudistomins Y_5_ **27**): Yellow solid; yield 66%; mp 267–268 °C; ^1^H NMR (600 MHz, DMSO-*d*_6_) δ 12.04 (s, 1H), 10.96 (s, 1H), 8.57 (d, *J* = 5.5 Hz, 1H), 8.54 (s, 2H), 8.47 (d, *J* = 5.5 Hz, 1H), 8.33 (d, *J* = 7.3 Hz, 1H), 7.81 (d, *J* = 8.2 Hz, 1H), 7.61 (t, *J* = 7.3 Hz, 1H), 7.32 (t, *J* = 7.3 Hz, 1H);^ 13^C NMR (151 MHz, DMSO-*d*_6_) δ 188.9, 154.5, 141.7, 137.1, 136.0, 135.9, 135.4, 131.2, 131.0, 129.0, 121.9, 120.3, 120.0, 119.1, 113.0, 110.9; HRMS: *m/z* calcd. for C_18_H_9_N_2_O_2_Br_2_^−^, 442.9031; found: 442.9030.

(6-Bromo-9*H*-pyrido[3,4-*b*]indol-1-yl)(3,5-dibromo-4-hydroxyphenyl)methanone (Eudistomins Y_6_ **28**): Yellow solid; yield 62%; mp 277–278 °C; ^1^H NMR (600 MHz, DMSO-*d*_6_) δ 12.16 (s, 1H), 10.97 (brs, 1H), 8.61 (s, 1H), 8.59 (d, *J* = 4.4 Hz, 1H), 8.52 (s, 2H), 8.51 (d, *J* = 4.4 Hz, 1H), 7.76 (d, *J* = 8.8 Hz, 1H), 7.73 (dd, *J* = 8.8, 2.2 Hz, 1H);^ 13^C NMR (151 MHz, DMSO-d_6_) δ 188.8, 154.6, 140.4, 137.4, 136.4, 136.0, 135.4, 131.5, 130.8, 130.1, 124.5, 122.0, 119.6, 115.0, 112.3, 110.9; HRMS: *m/z* calcd. for C_18_H_8_N_2_O_2_Br_3_^−^, 520.8136; found: 520.8134.

(7-Bromo-9*H*-pyrido[3,4-*b*]indol-1-yl)(3,5-dibromo-4-hydroxyphenyl)methanone (Eudistomins Y_7_ **29**): Yellow solid; yield 62%; mp 297–298 °C; ^1^H NMR (600 MHz, DMSO-*d*_6_) δ 12.11 (s, 1H), 10.96 (brs, 1H), 8.58 (d, *J* = 4.6 Hz, 1H), 8.53 (s, 2H), 8.46 (d, *J* = 4.6 Hz, 1H), 8.27 (d, *J* = 8.2 Hz, 1H), 7.97 (d, *J* = 1.8 Hz, 1H), 7.45 (dd, *J* = 8.2, 1.8 Hz, 1H); ^13^C NMR (151 MHz, DMSO-*d*_6_) δ 188.8, 154.6, 142.5, 137.7, 136.3, 136.0, 135.4, 130.8, 130.6, 123.7, 123.2, 121.8, 119.2, 115.6, 110.8; HRMS: *m/z* calcd. for C_18_H_8_N_2_O_2_Br_3_^−^, 520.8136; found: 520.8141.

## 4. Conclusion

In summary, we reported the synthesis of the natural β-carboline alkaloid Eudistomins Y_1_–Y_7_ using the modified Pictet-Spengler reaction in a one-pot process. The preliminary biological activities of the target marine alkaloids and their methylated productions **16**–**22** were studied.
